# Determinants of lifestyle behavior in type 2 diabetes: results of the 2011 cross-sectional survey on living with chronic diseases in Canada

**DOI:** 10.1186/1471-2458-13-451

**Published:** 2013-05-07

**Authors:** Calypse B Agborsangaya, Marianne E Gee, Steven T Johnson, Peggy Dunbar, Marie-France Langlois, Lawrence A Leiter, Catherine Pelletier, Jeffrey A Johnson

**Affiliations:** 1 Department of Public Health Sciences, 2–040 Li Ka Shing Center for Health Research and Innovation, University of Alberta, Edmonton, AB T6G 2E1, Canada; 2 Centre for Chronic Disease Prevention and Control, Public Health Agency of Canada, 785 Carling Avenue, Ottawa, ON, K1A 0K9, Canada; 3 Centre for Nursing & Health Studies, Faculty of Health Disciplines, 1 University Drive, Athabasca University, Athabasca, Alberta, T9S 3A3, Canada; 4 Diabetes Care Program of Nova Scotia, Halifax, NS, B3H 2Y9, Canada; 5 Division of Endocrinology, Faculté de médecine et des sciences de la santé, Université de Sherbrooke and Étienne-LeBel Clinical Research Center, Centre Hospitalier Universitaire de Sherbrooke, Sherbrooke, QC, J1H 5N4, Canada; 6 Keenan Research Centre in the Li Ka Shing Knowledge Institute of St. Michael’s Hospital, Division of Endocrinology & Metabolism, University of Toronto, Toronto, Ontario, M5B 1W8, Canada

**Keywords:** Type 2 diabetes, Health behaviors, Health professional advice, Self-management

## Abstract

**Background:**

Lifestyle behavior modification is an essential component of self-management of type 2 diabetes. We evaluated the prevalence of engagement in lifestyle behaviors for management of the disease, as well as the impact of healthcare professional support on these behaviors.

**Methods:**

Self-reported data were available from 2682 adult respondents, age 20 years or older, to the 2011 Survey on Living with Chronic Diseases in Canada’s diabetes component. Associations with *never* engaging in and *not* sustaining self-management behaviors (of dietary change, weight control, exercise, and smoking cessation) were evaluated using binomial regression models.

**Results:**

The prevalence of reported dietary change, weight control/loss, increased exercise and smoking cessation (among those who smoked since being diagnosed) were 89.7%, 72.1%, 69.5%, and 30.6%, respectively. Those who reported not receiving health professional advice in the previous 12 months were more likely to report *never* engaging in dietary change (RR = 2.7, 95% CI 1.8 – 4.2), exercise (RR = 1.7, 95% CI 1.3 – 2.1), or weight control/loss (RR = 2.2, 95% CI 1.3 – 3.6), but not smoking cessation (RR = 1.0; 95% CI: 0.7 – 1.5). Also, living with diabetes for more than six years was associated with *not* sustaining dietary change, weight loss and smoking cessation.

**Conclusion:**

Health professional advice for lifestyle behaviors for type 2 diabetes self-management may support individual actions. Patients living with the disease for more than 6 years may require additional support in sustaining recommended behaviors.

## Background

Diabetes is a major public health concern around the world. In Canada, over two million adults lived with the disease in 2009 [1,2]. Considering that about 200,000 new cases are diagnosed yearly [1], the burden of diabetes is projected to increase [3]. It is also a major driver of the total health care cost and the sixth leading cause of death in Canada [4]. The vast majority (> 90%) of all cases are type 2 diabetes, diagnosed in older adults, and often associated with obesity [5].

Lifestyle behavior modification is a cornerstone for self-management of type 2 diabetes [6,7]. Important components of self-management include maintaining a healthy diet, participating in regular physical activity, achieving and maintaining a healthy body weight, limiting alcohol intake, and not/quitting smoking. Because the complexity of diabetes management requires that health professionals work collaboratively with their patients [8], self-management support has become a critical element for effective diabetes self-management.

Although there are published studies on effective self-management among Canadians living with type 2 diabetes [9-11], data from a large nationally representative sample may provide further evidence to inform strategies that will support long-term maintenance of self-management behaviors. To date, few population-based studies have described the self-management practices of Canadian adults with type 2 diabetes. Among those that have [12,13], the reported self-management behaviors are not consistent with guidelines for diabetes self-management [6]. A better understanding of these behavior patterns among persons living with the disease may provide vital information for public health planning.

In the present study, we set to 1) understand the extent to which Canadians living with type 2 diabetes employ different lifestyle behaviors for the management of the disease, by engaging in dietary change, exercise, weight control/loss, smoking cessation and limiting alcohol intake; 2) determine the proportion of persons with type 2 diabetes who receive healthcare professional advice and also engage in self-care behaviors; and 3) identify determinants of a) *never* engaging in these lifestyle behaviors, and b) *not* sustaining lifestyle behaviors for the management of type 2 diabetes.

## Methods

The study is based on data from the 2011 *Survey on Living with Chronic Diseases in Canada* Diabetes Component (SLCDC-DM). Individuals age 20 years and older who reported having diabetes diagnosed by a health care professional as part of the 2010 Canadian Community Health Survey (CCHS) were eligible for participation in the SLCDC-DM. Of the 3590 CCHS respondents contacted, 2933 individuals agreed to participate in the SLCDC-DM, resulting in a response rate of 81.7% [14]. Members of the Canadian Forces, First Nations individuals living on reserves, individuals residing in institutions, and residents of Canada’s three territories, Nunavut, Northwest Territories and Yukon Territory, and respondents who reported having diabetes only during pregnancy were excluded from the sampling frame [14]. Furthermore, respondents who reported having been diagnosed during pregnancy but did not state whether they had been diagnosed outside of pregnancy were excluded (n=7). The analysis was restricted to the population with self-reported type 2 diabetes; these were individuals who reported either having type 2 diabetes or did not report a type but reported being diagnosed after the age of 30 years.

The population represented in this survey was characterized by time since diagnosis (≤ 2 years, 3–5 years, 6+ years) and socio-demographic characteristics (age, gender, ethnicity, educational attainment, total household income, marital status, urban versus rural residence). Time since diagnosis was derived based on current age and responses to the question “How old were you when you were first diagnosed with diabetes?” With the exception of age, gender, and residence location, other information on socio-demographic characteristics was measured as part of the 2010 CCHS, which was linked to the 2011 SLCDC.

Participants were asked if, as a result of being diagnosed with diabetes, they *ever*: i) *changed the type or amount of food*, ii) *changed the amount of exercise or participated in physical activities*, and iii*) stopped drinking or limited alcohol intake*? Those who responded “yes” were further asked if they continued to maintain the change “all the time”, “most of the time*”, “*some of the time*”* or “none of the time” for dietary change, physical activity and alcohol intake. For our analysis, participants were categorized as *not* sustaining the self-management behavior change, if they reported “none of the time” or “some of the time”. Likewise, patients were also asked if, as a result of being diagnosed with diabetes*,* they ever: i) *tried to control or lose weight*, and ii) *quit smoking.* Patients who responded “yes” were further asked: *are you continuing to maintain this change*? Those who responded “no” were considered as not sustaining the self-management behavior change. Regular drinking of alcohol was defined as 14 standard drinks of alcohol/week for men or 9 standard drinks of alcohol/week for women [6].

Never engaging in lifestyle behaviors and not sustaining lifestyle behaviors were described according to whether the respondent reported having received self-management support (i.e. advice for the behavior from a health care professional) in the previous 12 months. For example, participants were asked “*In the past 12 months, has a doctor or other health professional discussed changing the type or amount of food you eat to help you control your diabetes?”* Similar questions were asked for physical activity/exercise, controlling/losing weight, quitting smoking, and limiting alcohol consumption.

The weighted prevalence of engaging in self-reported behaviors for type 2 diabetes management was estimated. Using cross tabulations, the weighted proportions of respondents engaging in self-management behaviors were estimated according to whether or not patients received health professional advice for lifestyle behaviors. Associations between descriptors and a) *never* engaging in lifestyle behaviors, and b) *not* sustaining lifestyle behaviors were examined using multivariate prevalence rate ratios (RRs), estimated using log-binomial regression models. Data were analyzed using SAS Enterprise Guide version 4 (Cary, NC). Point estimates were weighted to reflect the Canadian adult population, with population estimates based on 2006 Census counts and counts of birth, death, immigration and emigration since that time [14,15]. To account for stratification and clustering in the SLCDC design, 95% confidence intervals (CI) were calculated using exact standard errors generated through bootstrap re-sampling methods [16].

Informed consent was obtained from all survey respondents. All personal information created, held or collected by Statistics Canada is protected by the Privacy Act and by the Statistics Act. Share partners, including the Public health Agency of Canada, have access to the data under the terms of the data sharing agreements. These data files only contain information on respondents who agreed to share their data with Statistics Canada’s partners.

## Results

### Socio-demographic characteristics

Of the 2933 respondents to the survey, 2682 (91.4%) who reported type 2 diabetes were included in these analyses, excluding those with type 1 diabetes mellitus. The total sample was based on either their self-reported type 2 diabetes (n=2351) or age at diagnosis ≥ 30 years (n=331). The socio-demographic characteristics of the study population are presented in Table 1. The respondent sample was predominantly male (58.1%), living in urban regions (82.4%), and almost half were diagnosed over ten years ago (44.9%).

**Table 1:** Characteristics of the study population in the 2011 survey on living with chronic disease in Canada (N=2,682)

	**N**	% (95% CI)*
Gender		
Female	1338	41.9 (39.1, 44.8)
Male	1344	58.1 (55.2, 60.9)
Age (years)		
20-44	118	6.6 (4.9, 8.4)
45-64	1003	45.7 (42.5, 49.0)
65+	1561	47.6 (44.6, 50.6)
Mean (standard error) = 63.4 (0.4)		
Ethnicity		
White	2428	81.0 (77.8, 84.2)
Aboriginal off-reserve	101	3.3 (2.3, 4.3)
Other	142	15.7 (12.5, 19.0)
*Missing = 11*		
Education		
Less than secondary	851	26.0 (23.3, 28.6)
Secondary school graduate	431	16.7 (14.2, 19.2)
Some post-secondary education	176	7.6 (5.8, 9.5)
Post-secondary graduate	1208	49.7 (46.5, 52.9)
*Missing = 16*		
Total household income		
<$15,000	256	7.1 (5.6, 8.6)
$15,000-$29,999	648	19.5 (17.0, 21.9)
$30,000-$49,999	637	25.0 (22.1, 27.9)
$50,000-$79,999	501	25.5 (22.3, 28.6)
≥ $80,000	368	23.0 (19.8, 26.1)
*Missing = 272*		
Marital status		
Married/common-law	1475	69.5 (66.9, 72.2)
Widowed/separated/divorced	921	22.1 (19.8, 24.4)
Single	284	8.4 (6.8, 10.1)
*Missing = 2*		
Region**‡**		
Urban	2126	82.4 (79.9, 85.0)
Rural	556	17.6 (15.0, 20.1)
Time since diagnosis		
≤2 years	396	15.4 (12.9, 17.8)
3–5 years	549	21.9 (19.1, 24.6)
6+	1718	62.8 (59.5, 66.0)
*Missing = 19*		

* Proportions are weighted to reflect the Canadian adult population.

**‡ **Region of residence was determined based on postal code. Urban areas are those continuously built-up areas having a population concentration of 1,000 or more and a population density of 400 or more per square kilometer based on 2006 Census population counts.

### Prevalence of lifestyle behaviors

Since being diagnosed with diabetes, 89.7% of the respondents reported changing the type or amount of food they ate; 69.5% increased their amount of exercise or participation in physical activity; 55.6% were trying to control or lose weight. Among those who drank more than 9 alcoholic drinks/week for women and 14 drinks/week for men at diagnosis, 42.8% reported that they reduced their alcohol intake.

Overall, less than half reported engaging in each of the lifestyle behaviors *all of the time*: 37.0% for diet; 18.0% for physical activity or exercise; 39.8% for weight control; 30.6% for smoking cessation; and 17.7% for limiting alcohol. Among those who reported smoking at diagnosis (23.8%), half (50.5%) never quit and 18.9% tried to quit, but had not sustained the change (Table 2).

**Table 2:** Frequency of self-management behaviors among Canadian adults age 20 years and older living with type 2 diabetes following diagnosis

**Frequency of behavior**	**Overall**		
	**N**	**%**	**(95%CI)**
Diet			
All the time	952	37.0	(34.0, 40.1)
Most of the time	1112	38.1	(35.0, 41.2)
Some of the time	255	12.2	(9.8, 14.6)
Already doing so for other reasons*	62	2.3†	(1.4, 3.2)
No longer does so	**‡**	**‡**	
Never did so	279	10.1	(8.3, 12.0)
Exercise or physical activities			
All the time	505	18.0	(15.6, 20.4)
Most of the time	573	22.8	(20.0, 25.7)
Some of the time	349	13.0	(10.8, 15.3)
Already doing so for other reasons*	424	15.6	(13.1, 18.1)
No longer does so	63	2.8 ^E^	(1.7, 4.0)
Never did so	762	27.6	(25.0, 30.3)
Control or lose weight			
Achieved/maintained goal weight	1017	39.8	(36.6, 42.9)
Still trying to control/lose weight	421	14.7	(12.4, 17.0)
Already doing so for other reasons*	43	1.1†	(0.6, 1.6)
Not doing so because already a healthy weight	461	16.5	(14.1, 19.0)
No longer does so	503	20.2	(17.5, 22.9)
Never did so	217	7.7	(5.9, 9.5)
Quit smoking (Among smokers, n= 626)			
Quit smoking, sustained	190	30.6	(24.5, 36.5)
Quit smoking, started again	99	18.9	(13.4, 24.4)
Never quit smoking	334	50.5	(43.8, 57.2)
Limits alcohol consumption (among those who reported drinking more than 9 drinks per week for women or 14 drinks per week for men since diagnosis n=191)			
All the time	49	17.7†	(9.1, 26.3)
Most of the time	39	25.1 †	(13.3, 37.0)
Some of the time	**‡**	**‡**	
No longer does so	0	0	
Never did so	91	45.7	(32.3, 59.1)

**†- **Interpret with caution (coefficient of variation 16.6% to 33.3%).

‡ - Too unreliable to be reported (coefficient of variation > 33.3% or n<30).

* Individuals who reported never or no longer engaging in the behaviour for diabetes management were asked to specify the reason for not doing so. These individuals reported that they were already engaging in the behaviour for other reasons beyond diabetes management.

Participants who reported having discussed behavior change with a health professional were more likely to report behavior modification compared to those who did not report receiving advice for diet (95.7% vs. 85.0% p < 0.0001), physical activity (79.6% vs. 60.7%, p < 0.0001), weight loss (94.5% vs. 88.9%, p < 0.002), and limiting alcohol consumption (among those who reported drinking alcohol, 74.6% vs. 40.2%, p = 0.04) (Table 3).

**Table 3:** Proportion of Canadian adults age 20 years or older with type 2 diabetes (n=2,682) who engage in self-management behaviors by receipt of health professional advice for the behavior

	**Ever engaged in respective self-management behavior change**		
**Health professional advice received (past 12 months)**	**n**	**% (95% CI)**	**P value**
Diet			
Yes (n=1141)	1071	95.7 (94.2, 97.1)	
No (n=1534)	1320	85.0 (81.8, 88.1)	P < 0.0001
*Missing = 7*			
Physical activity or exercise			
Yes (n=1618)	1263	79.5 (76.4, 82.7)	
No (n=1056)	645	60.7 (55.8, 65.6)	P < 0.0001
*Missing = 8*			
Weight control			
Yes (n=1466)	1383	94.7 (92.7, 96.7)	
No (n=1196)	1055	88.9 (85.7, 92.1)	P = 0.002
*Missing =20*			
Quitting smoking (among smokers, n=509)			
Yes (n=331)	132	43.3 (34.5, 52.2)	
No (n=122)	33	36.6 (18.5, 54.7) *	P = 0.5
*Missing = 56*			
Limiting alcohol consumption (among those who reported drinking more than 9 drinks per week for women or 14 drinks per week for men in the previous 12 months n=102)			
Yes (n=33)	‡	74.6 (49.7, 99.6) *	
No (n=57)	‡	40.2 (19.2, 61.1) *	P = 0.04
*Missing = 12*			

*- Interpret with caution (coefficient of variation 16.6% to 33.3%).

‡ - Too unreliable to be reported (coefficient of variation > 33.3% or n<30.

### Associations with *never* engaging/*not* sustaining lifestyle behaviors

In multivariate analysis, an increased prevalence of *never* changing their diet was associated with being male (RR = 1.9, 95% CI 1.3 – 2.8), age ≥ 65 years (RR = 2.6, 95% CI 1.6 – 4.2), or having less than secondary education (RR = 1.7, 95% CI 1.2 – 2.4) (Table 4). Compared to those with total household income ≥ 80,000 CAD, individuals with lower income levels were more likely to report *never* having changed their diet (RRs ranging from 3.8 to 4.5) or never engaged in exercise (RRs ranging from 1.6 to 2.1). Also, not receiving advice for the specific self-management behavior from health professionals in the previous 12 months was associated with *never* engaging in lifestyle behavior for: diet (RR = 2.7, 95% CI 1.8 – 4.2), exercise (RR = 1.7, 95% CI 1.3 – 2.1), or controlling/losing weight (RR = 2.2, 95% CI 1.3 – 3.6).

**Table 4:** **Multivariate associations between individual characteristics and *never *engaging in self-management behaviors among Canadian adults age 20 years and older with type 2 diabetes (n=2,682)**

	**Never changed diet**	**Never engaged in exercise**	**Never controlled/lost weight**	**Never quit smoking**
	**Multivariate RR**	**Multivariate RR**	**Multivariate RR**	**Multivariate RR**
Age				
20-64	1	1	1	1
65+	2.6 (1.6, 4.2)*	0.9 (0.7, 1.2)	1.5 (0.9, 2.5)	1.1 (0.3, 1.4)
Sex				
Female	1	1	1	1
Male	1.9 (1.3, 2.8)*	1.0 (0.8, 1.2)	1.5 (1.0, 2.4)	1.0 (0.7, 1.3)
Education				
Post-secondary graduate	1	1	1	1
Some post-secondary	1.7 (0.8, 3.8)	0.8 (0.5, 1.3)	0.9 (0.3, 2.6)	0.8 (0.4, 1.7)
Secondary graduate	0.9 (0.4, 1.8)	0.8 (0.6, 1.1)	0.8 (0.4, 1.6)	0.9 (0.6, 1.4)
< Secondary	1.7 (1.2, 2.4)*	1.3 (1.1, 1.7)*	0.8 (0.5, 1.3)	0.9 (0.7, 1.2)
Income				
≥ $80,000	1	1	1	1
$50,000-$79,999	4.0 (1.7, 9.2)*	1.3 (0.8, 2.1)	1.0 (0.4, 2.5)	0.9 (0.5, 1.8)
$30,000-$49,000	3.8 (1.8, 8.1)*	1.6 (1.0, 2.4)*	1.0 (0.4, 2.4)	0.9 (0.5, 1.5)
$15,000-$29,999	4.1 (1.9, 8.6)*	2.1 (1.3, 3.6)*	1.0 (0.5, 2.3)	1.0 (0.6, 1.8)
<$15,000	4.5 (1.9, 10.6)*	2.0 (1.2, 3.5)*	2.1 (0.8, 5.7)	0.7 (0.4, 1.3)
Time since diagnosis				
< 2 years	1	1	1	1
3–5 years	0.9 (0.4, 1.5)	1.4 (1.0, 2.1)	1.1 (0.5, 2.5)	0.7 (0.4, 1.2)
6+ years	0.9 (0.5, 1.4)	1.2 (0.8, 1.7)	0.8 (0.4, 1.7)	0.7 (0.5, 1.2)
Ethnicity				
White	1	1	1	1
Aboriginal off-reserve	1.6 (0.8, 3.3)	0.9 (0.5, 1.6)	0.9 (0.4, 2.1)	1.7 (0.9, 3.2)
Other	3.3 (1.7, 6.3)*	0.7 (0.4, 1.2)	1.7 (0.8, 3.8)	0.5 (0.1, 2.0)
Married/common-law				
No	1	1	1	1
Yes	1.0 (0.6, 1.5)	1.2 (0.9, 1.5)	1.1 (0.5, 1.2)	0.6 (0.4, 1.0)*
Region				
Rural	1	1	1	1
Urban	0.8 (0.5, 1.3)	0.9 (0.7, 1.1)	1.1 (0.7, 1.8)	0.8 (0.5, 1.1)
Advice for behavior provided				
Yes	1	1	1	1
No	2.7 (1.8, 4.2)*	1.7 (1.3, 2.1)*	2.2 (1.3, 3.6)*	1.0 (0.7, 1.5)

*Significant estimates (p<0.05).

Among those who reported *ever* engaging in diabetes self-management lifestyle behavior change, living with diabetes for 6 or more years was associated with *not* sustaining diet change (RR = 2.0, 95% CI 1.2 – 3.4), weight loss (RR = 1.8, 95% CI 1.2 – 2.6), and smoking cessation (RR = 3.8, 95% CI 1.0 – 13.9). Similar findings were observed for persons who had lived with diabetes for over 10 years (Table 5). No differences were noted in our findings after adjusting for patients’ use of support services or programs.

**Table 5:** **Multivariate associations between individual characteristics and *not *sustaining lifestyle behaviors for diabetes management, among Canadian adults age 20 years and older with type 2 diabetes who ever engaged in the lifestyle behaviors**

	**Not sustained diet change**	**Not sustained exercise engagement**	**Not sustained weight control**	**Not sustained smoking cessation**
	**Multivariate RR**	**Multivariate RR**	**Multivariate RR**	**Multivariate RR**
Age				
20-64	1	1	1	1
65+	0.5 (0.3, 0.6)*	0.9 (0.8, 1.1)	0.8 (0.6, 1.1)	0.9 (0.5, 1.4)
Sex				
Female	1	1	1	1
Male	0.7 (0.4, 1.0)	1.1 (0.9, 1.3)	0.9 (0.6, 1.1)	1.0 (0.6, 1.7)
Education				
Post-secondary graduate	1	1	1	1
Some post-secondary	1.1 (0.6, 2.0)	1.0 (0.7, 1.5)	1.0 (0.6, 1.6)	1.9 (0.8, 4.6)
Secondary graduate	0.9 (0.5, 1.5)	0.9 (0.7, 1.2)	1.2 (0.8, 1.7)	0.9 (0.4, 1.7)
< Secondary	1.0 (0.7, 1.6)	1.2 (1.0, 1.5)	1.0 (0.7, 1.4)	1.0 (0.6, 1.7)
Income				
≥ $80,000	1	1	1	1
$50,000-$79,999	1.4 (0.8, 2.5)	1.3 (0.9, 1.8)	1.1 (0.7, 1.7)	1.0 (0.4, 2.4)
$30,000-$49,000	0.7 (0.4, 1.2)	1.2 (0.9, 1.7)	0.8 (0.5, 1.3)	0.8, 0.3, 2.2)
$15,000-$29,999	1.1 (0.6, 2.1)	1.0 (0.7, 1.3)	1.0 (0.6, 1.7)	1.2 (0.5, 3.1)
<$15,000	1.0 (0.5, 2.2)	1.0 (0.6, 1.5)	0.8 (0.4, 1.6)	1.0 (0.3, 3.1)
Time since diagnosis				
< 2 years	1	1	1	1
3–5 years	1.6 (0.9, 3.0)	1.0 (0.7, 1.4)	1.2 (0.8, 2.0)	3.2 (0.8, 12.8)
6+ years	2.0 (1.2, 3.4)*	1.1 (0.9, 1.4)	1.8 (1.2, 2.6)*	3.8 (1.0, 13.9)*
Ethnicity				
White	1	1	1	1
Aboriginal off-reserve	1.4 (0.6, 3.2)	0.7 (0.4, 1.2)	1.3 (0.6, 2.6)	1.2 (0.2, 5.8)
Other	1.2 (0.6, 2.7)	0.8 (0.5, 1.4)	0.9 (0.5, 1.8)	0.6 (0.1, 5.1)
Married/common-law				
No	1	1	1	1
Yes	1.1 (0.7, 1.7)	0.9 (0.8, 1.1)	1.1 (0.8, 1.5)	1.3 (0.7, 2.4)
Region				
Rural	1	1	1	1
Urban	0.8 (0.5, 1.3)	1.0 (0.8, 1.2)	0.9 (0.7, 1.3)	1.4 (0.6, 3.0)
Advice for behavior provided				
Yes	1	1	1	1
No	0.7 (0.5, 1.1)	1.2 (1.0, 1.5)*	0.3 (0.2, 0.5)*	0.5 (0.1, 1.8)

*Significant estimates (p<0.05).

## Discussion

In this study describing the self-reported self-management behaviors in a nationally representative sample of adult Canadians living with type 2 diabetes, we observed that lifestyle behavior change (in particular dietary change, exercise and weight control) appeared to be prevalent following a diagnosis, especially among patients who receive self-management advice from a health professional. However, smoking cessation may represent a challenge; only 31% of those who reported having smoked after diagnosis reported having successfully quit. Among persons who ever engaged in lifestyle behaviors for self-management, persons living with diabetes for over 6 years were more likely to *not* sustain the behaviors.

The finding that persons who do not receive health professional advice for behavior change are less likely to engage in diabetes self-management behaviors is consistent with previous findings [17,18]. Individuals with type 2 diabetes who receive self-management support from physicians, nurses, pharmacists, dieticians or other health professionals on the management of their diet [18], exercise and weight management [19] or combinations thereof [20] are generally more likely to make such changes. In a randomized control trial of patients with type 2 diabetes [19], a brief intervention to increase dialogue between patients and health care providers about lifestyle behavior modification for diabetes self-management significantly improved the level of recommended physical activity and weight loss. Our findings highlight the importance of health care provider communication, either through the provision of information or participatory decision-making, in patients’ behaviors for diabetes self-management. Better healthcare provider communication may lead to better diabetes self-management and, in turn, improve health outcomes, including higher levels of patient satisfaction [21-23]. Nonetheless, it is important to also note that substantial numbers of individuals who reported not receiving advice from health professional for change in diet, exercise or weight still engaged in self-management behaviors, and may have accessed support services or programs independent of health professional advice. Whether these individuals were already living healthy lifestyles and are less likely to receive health professional advice could not be addressed with these data and therefore remains to be studied.

The prevalence of smoking among adults with type 2 diabetes in this study is comparable to previous reports in adult Canadians living with diabetes [1] and it is interesting that no difference was observed in the proportion of respondents who ever engaged in smoking cessation in our multivariate analyses. The null effect of healthcare provider dialogue on smoking cessation observed in this study highlights the need for a more rigorous smoking cessation program for diabetes care, apart from standard or usual care self-management education in persons with diabetes [24,25]. The American Diabetes Association, for instance, recommends healthcare providers to utilize more intensive interventions for smoking cessation as a priority of care for diabetic smokers [7]. Because smoking exacerbates the risk of developing both macrovascular and microvascular complications in patients with diabetes [26], the Canadian Diabetes Association Clinical Practice Guidelines asserts that “the first priority” in the prevention of diabetes complications should be the reduction in the risk for cardiovascular disease through a multifaceted, multi-factorial, approach, including smoking cessation [6].

Persons with lower household income were more likely to report never changing their diet or not engaging in exercise for self-management of type 2 diabetes. Socio-economic status (SES) is an important determinant of diabetes self-management behaviors, particularly diet and exercise. Socioeconomic disadvantage is associated with a wide range of risk behaviors, which in turn have a negative consequence on health [27]. It is possible that this relationship is mediated by poor adherence to diabetes self-management recommendations, which is related to low accessibility to healthy foods and health promotion facilities. Elimination of disparities in health, through policies and interventions to improve access and quality of care in patients with type 2 diabetes should be prioritized.

We observed that persons living with diagnosed type 2 diabetes for more than 6 years are more likely to *not sustain* lifestyle behavior changes for diabetes self-management. In another Canadian study [28], self-management education was shown to have a significant impact in healthy eating among diabetes patients, and was sustained at 2 years. The study did not, however, capture the self-management behavior sustenance for more than 2 years. Our results indicate that persons living with type 2 diabetes for more than 6 years may need specific attention to maintain behavior change. Further studies are necessary to understand barriers to sustained self-management behavior change among patients living with the disease for longer durations.

The Survey on Living with Chronic Diseases in Canada is a population-based cross-sectional survey designed to provide information on chronic disease management among Canadians living with chronic diseases. Several limitations are noted. First, the survey was insufficiently powered to assess the effect of ethnicity on engagement in lifestyle behaviors. Because multiple ethnic populations known to be culturally and epidemiologically heterogeneous [29] were combined as a single category, differences in diabetes self-management behavior among these populations may have been masked. Second, it is acknowledged that self-reported adherence to recommended lifestyle behaviors can be overestimated in self-reported surveys due to social desirability. Our findings nevertheless provide an important profile of the relative prevalence of self-reported lifestyle behaviors for managing type 2 diabetes. Likewise, participants may not accurately remember the healthcare services they received from their healthcare providers; research has shown that a patient’s ability to retain information from health professionals is often limited, with 40-80% of information forgotten immediately [30,31].

Third, the survey explores a limited range of determinants of lifestyle behaviors, and did not capture specific measures of lifestyle behavior change, e.g. patients’ self-reported engagement in physical activity was captured, without reference to the exercise intensity. The use of direct measures of physical activity (such as accelerometry), which would provide a more accurate estimate of physical activity levels including the intensity, was not feasible for this large national survey. On another note, never engaging and not sustaining lifestyle behaviors were described according to whether the respondent reported having received advice for the behavior from a health care professional. It is certainly possible that some patients changed their lifestyle behaviors on their own, with the use of self help books, internet, or other sources without having received specific advice from a health care professional [32].

This study may have some implications for action. First, although there is a high prevalence of diabetes self-management behavior modification among people living with type 2 diabetes in Canada, the findings suggest a need for more action. Receipt of advice from health care professionals is strongly associated with self-reported engagement in self-management behaviors. Of note, however, is the limited effect of dialogue on smoking cessation among patients with type 2 diabetes that smoke. This, perhaps, indicates a need for more aggressive smoking cessation interventions. Finally, the study notes that SES-dependent disparities in healthy lifestyle behaviors are observed among Canadians living with type 2 diabetes.

## Conclusion

In conclusion, this study shows that among adult Canadians living with type 2 diabetes, self-management behavior change is common, particularly among newly diagnosed individuals and those receiving lifestyle behavior change advice from health professionals. More action is needed for improved diabetes self-management among those living with the disease for longer durations, those with lower socioeconomic status, and those patients who continue to smoke. Provision of information and advice for lifestyle behavior at the primary care level, and targeted to these higher risk groups, may be an effective health promotion strategy.

## Competing interests

The authors declare that they have no competing interests.

## Authors’ contribution

CBA: Conception and design, interpretation of data, drafting of manuscript and revision of manuscript. MEG: Data acquisition, conception and design, data analysis, critical revision of manuscript. STJ, PD, MFL and LAL: Conception and design, interpretation of data and critical revision of manuscript. CP: Data acquisition, conception and design and critical revision of manuscript. JAJ: Conception and design, interpretation of data and critical revision of manuscript. All authors read and approved the final manuscript.

## Pre-publication history

The pre-publication history for this paper can be accessed here:

http://www.biomedcentral.com/1471-2458/13/451/prepub
